# A qualitative exploration of the views of paramedics regarding the use of dark humour

**DOI:** 10.29045/14784726.2024.12.9.3.37

**Published:** 2024-12-01

**Authors:** Jennifer Mercer, Deborah Morgan, Robyn Lotto

**Affiliations:** North West Ambulance Service ORCID iD: https://orcid.org/0009-0004-5515-5533; Liverpool John Moores University ORCID iD: https://orcid.org/0009-0006-4751-8300; Liverpool John Moores University ORCID iD: https://orcid.org/0000-0002-4305-0513

**Keywords:** dark humour, paramedicine, qualitative

## Abstract

**Introduction:**

Dark humour, often known as black comedy or gallows humour, is a distinct kind of humour that explores subjects that are generally taboo or uncomfortable. Identifying the function and impact of black humour is important given the prevalence of life-or-death situations, crucial clinical judgements and emotionally charged situations in the pre-hospital environment. The primary aim is to investigate the world of dark humour within the setting of experienced paramedics.

**Methods:**

A qualitative approach was employed. Ten paramedics, each with over 24 months’ experience, were recruited via word of mouth and snowball sampling. Semi-structured interviews were conducted between October 2023 and January 2024, and data were studied using thematic analysis.

**Results:**

Four themes were identified: the perceptions of dark humour from the public, students and colleagues; the use of dark humour in building resilience and improving perseverance; the negatives to mental health of prolonged use of black humour; and the benefits of humour use to develop camaraderie within the ambulance service.

**Conclusion:**

The study explored the views of a small sample of paramedics on this subject. Dark humour was identified as both a beneficial coping mechanism for resilience and a means of fostering camaraderie. Conversely, a chronic reliance on dark humour can have negative mental health implications. Utilising the usage of coping mechanisms such as dark humour as a yardstick to measure mental well-being could be an important first step in adopting a more holistic approach to mental health within the paramedic community.

## Introduction

Dark humour, often referred to as black comedy or gallows humour, is a unique form of comedy that delves into topics that are typically considered taboo or uncomfortable (Coogan & Mallett, 2013). This genre of humour finds its comedic element in subjects that may be tragic, sensitive or morbid. It pushes the boundaries of what is socially acceptable, and this provocative approach is both fascinating and polarising (Vosmer, 2023).

Dark humour is characterised by its willingness to address severe and often painful subjects, such as death, disease, violence or societal taboos, with a comedic twist (Dehon et al., 2021). It allows individuals to create a psychological distance from their problems and temporarily escape the harsh realities of life (Rojas et al., 2022). By finding humour in the face of adversity, they may better manage their emotional responses and manage stress (Plester, 2009).

Dark humour, however, is not for everyone. What one person finds humorous, another may find offensive or distressing. The line between dark humour and outright insensitivity can be thin, and individuals must exercise discretion when using it, especially in a public-facing, professional capacity. Additionally, the use of humour, dark or otherwise, can be a deflective technique which, in some cases, can lead to maladaptive coping especially if relied upon chronically (Barwick, 2012; Danielson et al., 2021).

Humour is a universal coping mechanism that can help individuals deal with difficult situations. To date, the topic of dark humour in the UK emergency services has received limited attention from researchers. While some studies have explored the broader use of humour in coping with stress (Coogan & Mallett, 2013), few have delved explicitly into the nature and impact of dark humour in this context (Charman, 2013; Lancaster & Phillips, 2021; Williams, 2013).

### Aims and objectives

The primary aim of this study is to explore the use and perception of black humour within the context of experienced paramedics.

## Methods

A qualitative approach was adopted to provide insights into the views of 10 paramedics. Three semi-structured interviews were conducted between October 2023 and January 2024, comprising a maximum of four participants to allow for a well-flowing conversation and discussion (Supplementary 1).

### Participants and setting

To be included in the study, individuals had to be Health and Care Professions Council (HCPC)-registered, qualified paramedics who had experienced operational ambulance employment in the past 12 months. Ten paramedics, each with over 24 months’ experience, were recruited through word of mouth and snowball sampling. Recruitment originated from advertising on the Liverpool John Moores University module notice board and allowed for further participants to contribute through word of mouth.

Data collection was conducted virtually via Microsoft Teams^TM^ for pragmatic reasons, such as accessibility and convenience.

### Data collection

Consent forms, pre-designed questions, which were open in nature to allow for inductive approach, and information sheets were sent to participants two weeks before their requested interview date, allowing for any questions or concerns that may arise to be answered thoroughly. This ensured that all discussions within the semi-structured interviews were free from surprises and any triggering conditions.

Along with the author, two interviews each had four participants and a third interview had two individuals, with each interview lasting around one hour, to not over-saturate the discussion. The conversations ran freely, with the advantages of semi-structured questioning proving their benefits by allowing openness, while ensuring a sense of structure by the author to guide the exchange.

### Data analysis

The Microsoft Teams^TM^ transcription feature automatically scripted the discussions. For the accuracy of the data set, each transcript was reviewed and corrected by comparing it to the original video recording, producing an exact duplicate of the interviews. Two individuals read the texts separately to guarantee that the information produced by the auto-transcription function was an accurate record. Following this, the transcript data was cleaned, with any unnecessary or unrelated conversations removed, and it was then analysed using Braun and Clarke’s (2019) thematic method of analysis. This is a preferred method when analysing the data collected from semi-structured interviews, as it is widely used in various disciplines to uncover and interpret meanings, providing a rich and nuanced understanding of a subject (Braun & Clarke, 2019).

## Results

Three separate interviews were conducted within a two-month timeframe, due to shifts and studying constraints. Four overarching themes emerged from the data: perceptions, resilience and perseverance, negatives to mental health and camaraderie (Supplementary 2). Each section is represented with quotes from the original transcripts, identified by Participant 1 to Participant 10.

### Perceptions

The opening question, ‘What does the term “dark humour” mean to you?’, sparked the conversation and identified the first theme ‒ perceptions ‒ especially the negative associations that dark humour presents. A common response was anxiety that patients who overhear statements may infer a lack of empathy and compassion, or indifference to the patient’s health or family concerns. However, positive opinions encompassed the gratitude patients and families can obtain from medical professionals displaying humour and light-heartedness in a grief-stricken situation (Reay et al., 2018), with recurring code words being ‘reducing family grief’, ‘bringing humour into grief’ and ‘taking their minds off it’. However, it was essential to recognise the atmosphere of each situation, with other repetitive code words and comments identified such as ‘reading the room’ and ‘knowing your audience’. Participants’ responses reflected a keen awareness of the nuances of engaging in dark humour:

*Appropriately inappropriate for the correct audience.* (Participant 5)

*You have to be in the situation once or twice to appreciate the joke.* (Participant 3)

*Not funny for non-medical professionals.* (Participant 2)

A sub-theme that emerged within the wider discussion of perceptions was that of a perceived change in paramedic students’ personalities and emotional maturity since the pandemic. It was noted that the ‘COVID generation’ has poor social skills because most lectures and seminars were held online, with students unable to socialise with anybody outside of their class. Interviewees reported that these reduced social skills and somewhat sheltered university experience have created a cohort of physically and emotionally younger students, who have trouble accepting constructive criticism and social commentary and are naive and easily offended:

*I’ve noticed a reduced emotional maturity in students.* (Participant 1)

*COVID generation.* (Participant 4)

*Isolation and quarantine wouldn’t have helped their social skills.* (Participant 1)

*Reduced time on operational placements due to COVID . . . reduced exposure to our humour.* (Participant 6)

### Resilience and perseverance

The second theme represents the building of resilience and perseverance in both positive and negative ways. The use of dark humour as a coping skill was a consistent topic among interviewees:

*It gets us through the day, especially seeing life-changing things.* (Participant 8)

*Not necessarily laughing at the exact situation but at the events around the periphery, to make it bearable.* (Participant 5)

*We see a lot of death and dying, laughing about it helps build resilience.* (Participant 6)

*Can feel a bit helpless for some patients with waiting times, so having a joke with colleagues about their jobs [patients] can help lighten the mood.* (Participants 10)

Furthermore, a significant number of the remarks made by the individuals who were interviewed indicated that the usage of comedy by emergency service workers is thought to be ‘taboo’ and centred on ‘experiences that are not normally considered humorous’. This highlights the significance of the utilisation of humour as a means of coping with difficult situations. For example, participants discussed the use of shared phrases to regularly attended stressful incidents or categories of patients, such as gardeners ‘kicking the bucket’ or patients who die by suicide as ‘hanging around for us’.

### Negative implications to mental health

The third theme was the negative implications to interviewees’ mental health with the long-term use of dark humour. Interviewees discussed how excessive use might begin to affect their emotional processing and impact life beyond the workplace:

*I notice you start detaching from personal experiences.* (Participant 6)

*You notice when you start feeling burnt out, there’s a lot of empathy fatigue.* (Participant 2)

*Makes a barrier . . . but covers up processing.* (Participant 8)

*Creeps into family and personal life.* (Participant 6)

Additionally, among participants there was an acknowledgement of the avoidance aspect of using coping strategies such as dark humour to deal with difficult situations:

*We know it will affect us in the future, yet we still show up for work.* (Participant 10)

*It’s like Pandora’s box ... it’ll get us eventually.* (Participant 6)

*We probably have PTSD ... will get us eventually.* (Participant 4)

### Camaraderie

The fourth theme identified was camaraderie. Participants in the interviews frequently mentioned that colleagues had similar personalities and senses of humour, which is beneficial in times of emotional distress or extended periods of difficulties in the workplace. Responses suggested that a particular sense of humour or appreciation of dark humour may be a shared characteristic of individuals drawn to the profession:

*We all have similar personalities to understand the humour.* (Participant 2)

*A specific characteristic that the profession attracts.* (Participant 7)

*We all have the same sense of humour.* (Participant 9)

*We’ve all experienced it ... can lean on each other.* (Participant 1)

. . .* like a family . . .* (Participant 3)

## Discussion

This study confirmed a variety of stressors, such as occupational, organisational and relational issues, that have a continual and significant impact on the mental health and well-being of paramedics. Paramedics utilise a range of strategies that allow them to adapt, manage and successfully respond to the demands of their employment, one of which is the use of dark humour.

The perceptions of the use of dark humour by colleagues, students and the public were an important consideration for the participants. Paramedics, with experience, develop intuition regarding the public and the highly dynamic circumstances in their interactions, especially the volatile nature of stress, emotion and grief (Reay et al., 2018). It has been shown that emergency service workers have a high level of emotional intelligence, which can be used to their advantage when interacting with the public (Dilawar et al., 2019; Müller & van der Giessen, 2015; Nightingale et al., 2018; Risan et al., 2016) and knowing when to use and when to avoid the use of dark humour.

Conversely, the fear exists that the use of dark humour may suggest a lack of empathy or caring, or indifference to the patient’s condition or the family’s concerns, potentially leading to complaints, grievances or even referral to the HCPC (HCPC, 2023; Searle et al., 2017). Such concerns, particularly when identified in reflection after the fact, may increase stress levels and mitigate the potential benefits of the use of humour.

An unanticipated outcome of this study was the perceived difference in personalities of students who began their education through the COVID-19 pandemic. Various studies (Chen & Lucock 2022; Christopher 2015) have highlighted the increased mental health issues of UK students during the COVID-19 pandemic, with some suggesting that healthcare students bore the brunt of these increased mental health concerns, as they lost out on their university experience and, eventually, had to practise on the so-called ‘front line’ of the pandemic (Gadi et al., 2022). Future studies to compare the perceptions of dark humour and the choice of coping strategies in general among COVID-19 graduates and their more experienced colleagues would be helpful.

Regardless of the perception of the use of dark humour, this study suggests that the judicious use of it can be helpful in developing short-term resilience and perseverance, two hotly debated topics in an NHS, and particularly ambulance services, increasingly under pressure. 

Many of the comments made by the interviewees suggest that the humour practised by emergency service professionals helps alleviate the stress response to the traumatic incidents they are involved in, cementing the importance of its use as a coping mechanism. An informative study by Nelson et al. (2020), investigating the understanding of ambulance staff responding to deaths by suicide, noted that staff report difficulty in forgetting the events and that some can recall in fine detail the memories of the incidents attended. The comments made by the interviewees in the Nelson et al. (2020) study correlate to the comments made by the interviewees in this study, relating to the use of dark humour as a form of informal resilience and coping, but also more concerningly suggest that the clinicians did not formally or professionally process the scenes witnessed, which could lead to longer-term negative mental health sequelae.

Participants demonstrated insight into the possible future implications of deflecting feelings by using coping mechanisms such as dark humour. Interviewees identified concerns about the regular use of dark humour in lieu of appropriately processing grief, traumatic experiences, violent and depressing situations, particularly the dissociation during these situations, which they may or may not process later (Baqai, 2020; Beldon & Garside, 2022; Cocker & Joss, 2016; Regehr & Millar, 2007). Given the acknowledged higher prevalence of post-traumatic stress disorder (PTSD) among emergency service personnel, this is concerning. Additionally, although much of the research on the mental health of paramedics has focused on PTSD, this represents just one potential negative consequence of frequent exposure to traumatic and stressful situations. A comprehensive systematic review and meta-analysis (Petrie et al., 2018), looking at the prevalence of mental health conditions among ambulance personnel worldwide, found that although rates of PTSD are very high in this population, other mental health problems, such as depression and anxiety, are, in fact, more prevalent.

Additionally, participants identified that the use of dark humour amid regular exposure to traumatic incidents can impact their personal lives, affecting their relationships with families and partners. Study participants recognised the significance of family and friends in enhancing their ability to cope and develop resilience, a finding that aligns with prior reports from professionals in health and emergency services (Regehr, 2005; Regehr & Millar, 2007). Nevertheless, the individuals involved in this study seldom revealed distressing and emotionally charged elements related to their profession, which aligns with previous research by Regehr et al. (2023), indicating that paramedics employ a strategy of compartmentalisation to protect their loved ones from work-related concerns. Although well-intentioned, such compartmentalisation can create distance from supportive relationships within which processing and healing can begin (Isbell et al., 2020).

Throughout the interviews, members discussed ambulance service camaraderie. Freemantle et al. (2022) found that crewmates share emotions and that stressors can be discharged after gaining trust while working together. Lancaster & Phillips (2021) also note that camaraderie improves teamwork, since employees trust and rely on one another after experiencing diverse experiences and joking about it.

Establishing a positive rapport with colleagues was found to be a safeguard against the development of PTSD (Smeltzer et al., 2022). This discovery aligns with previous research conducted on emergency medical technicians (Sharp et al., 2022). Given the rigorous demands of the job, it is likely that ambulance staff will spend a significant portion of their time with their colleagues, potentially sacrificing time with their families. Undoubtedly, fostering a robust and thriving relationship with colleagues would significantly enhance mental well-being and productivity in the workplace. Developing strong camaraderie with co-workers can promote feelings of comfort, security, teamwork and coherence in the workplace. Over time, this could function as a catalyst for morale improvement, alleviate work-related stress, enhance job contentment and prevent the occurrence and advancement of PTSD among ambulance personnel (Fjeldheim et al., 2014; Koinis et al., 2015; van der Ploeg, 2003). A cross-sectional study conducted with a different type of emergency service, Australian volunteer firefighters, revealed that camaraderie, characterised by a sense of belonging, shared identity, reciprocal trust and strong positive ties among cohesive work groups, safeguarded against post-traumatic stress (Tuckey & Hayward, 2010). 

Although the findings of this study are insightful and important, further steps are necessary to translate this understanding of dark humour as both a risk factor and a protective factor in the mental health of paramedics into pragmatic workplace interventions. Further research might focus on analysing the relationship between use of dark humour and changing levels of burnout / workplace stress as measured by validated screening tools. This could lead to education/intervention initiatives harnessing the partnership/teamwork culture of paramedics to create an environment where teammates informally monitor each other’s level of dark humour usage as an early warning system for increasing burnout or other mental health conditions (Kleber, 2003; ALmutairi & El Mahalli, 2020).

### Limitations

The small sample size, consisting only of paramedics who are students or alumni of Liverpool John Moores University and who are completing or have completed their DipHE in Paramedic Practice, BSc in Paramedic Science or MSc in Advanced Clinical Practice, limits the research effort. Initially, the uniformity of the sample, consisting exclusively of Liverpool John Moores students, may create a bias in the research results, as the experiences and viewpoints of this group may not accurately reflect the broader community of paramedics. The participants’ common educational background may lead to a limited range of perspectives, which could restrict the applicability of the study’s findings to the broader paramedic community.

Expanding the research to encompass participants from other ambulance organisations, each with unique protocols, resources and challenges, would offer a more comprehensive perspective on paramedics’ emotional and professional encounters.

## Conclusions

This study builds on an increasing archive of research relating to the exploration and the use and perception of dark humour in the pre-hospital environment. Paramedics use dark humour as a coping mechanism in the increasingly challenging environment of emergency medical services.

Finding humour in the most difficult situations provides a therapeutic outlet for built-up stress and strengthens mental health in a job known for its demanding nature. In this setting, dark humour serves as an adaptive strategy, enabling paramedics to effectively negotiate the complex array of stressors inherent in their roles. Additionally, dark humour can catalyse camaraderie and strengthen teamwork under highly stressful circumstances.

Conversely, consistently depending on dark humour might result in a gradual disconnection from personal experiences, jeopardising connections with families and partners and contributing to negative mental health outcomes. Utilising coping mechanisms such as dark humour as a yardstick to measure mental well-being could be an important first step in the adoption of a more holistic approach to mental health within the paramedic community.

## Author contributions

JM was responsible for the study conception and design, data collection, transcription, coding, analysis and critical review of the submission. DM was responsible for interpretation of data, critical review and revision of the submission. RL was responsible for the study conception and design, analysis and critical review of the submission. JM acts as the guarantor for this article.

## Conflict of interest

None declared.

## Ethics

Approval was granted by Liverpool John Moores University research ethics committee, approval reference number NAHPGT(AHCP)2037.

## Funding

None.
